# A latent profile analysis of the link between sociocultural factors and health-related risk-taking among U.S. adults

**DOI:** 10.1186/s12889-021-10608-z

**Published:** 2021-03-19

**Authors:** Jessica K. Perrotte, Eric C. Shattuck, Colton L. Daniels, Xiaohe Xu, Thankam Sunil

**Affiliations:** 1 Department of Psychology, Texas State University, UAC 253, 601 University Drive, San Marcos, TX 78666 USA; 2 Institute for Health Disparities Research, University of Texas at San Antonio, One University Circle, MS 3.02.49, San Antonio, TX 78249 USA; 3 Department of Public Health, The University of Tennessee at Knoxville, 335 Claxton Complex, 1122 Volunteer Boulevard, Knoxville, TN 37996 USA; 4 Department of Sociology, University of Texas at San Antonio, One University Circle, San Antonio, TX 78249 USA; 5 School of Public Administration, Sichuan University, No.24 South Section I, Yihuan Road, Chengdu, 610065 China

**Keywords:** Health risk, Medical risk, Health behaviors, Gender, Gender role, Individualism, Collectivism, Familism, Locus of control

## Abstract

**Background:**

Research suggests that health/safety behaviors (e.g., drinking heavily) and medical behaviors (e.g., donating blood) may be perceived as inherently risky, and further suggests there is substantial variation in the likelihood of engaging in a particular health-related risk behavior across people. Research examining demographic and sociocultural factors related to both health/safety and medical risk-taking is highly limited. Importantly, with very few exceptions the literature examining health risks characterized by potentially hazardous health behaviors (e.g, heavy alcohol use, driving without a seatbelt) is kept separate from the literature examining health risks characterized by potentially beneficial medical behaviors (e.g., donating blood, taking medication). In the interest of health promotion, it is critical for researchers to identify – and describe – individuals who are less inclined to engage in health-harming behaviors while *at the same time* being more inclined to engage in health-benefiting behaviors. Identifying such a subtype of individuals was the guiding aim for this study.

**Method:**

A national sample of adults in the United States responded to a survey on sociocultural and demographic correlates of health behaviors. Health-related risk-taking indicators were measured using the items from the health/safety and medical subscales of the DOSPERT-M. Subtypes of risk-takers were identified using latent profile analysis (LPA). Follow-up analyses to describe subtype demographic characteristics were conducted.

**Results:**

LPA identified four subtypes of risk-takers, including a subtype (*n* = 565, 45% of the sample; labeled “divergent”) that was comprised of individuals who highly endorsed medical risk-taking (e.g., taking medicine, giving blood) and minimally endorsed health/safety risk-taking (e.g., drinking heavily, unprotected sex). Subsequent analyses suggested that, among other findings, divergent profile members were likely to be married, endorse familial interdependence, and orient toward masculinity rather than femininity.

**Conclusion:**

By examining potentially modifiable factors related to individuals’ inclinations to engage in health protective behaviors, this study is an important step toward improving current health behavior interventions among U.S. adults.

Multiple possible outcomes are associated with any given health behavior and this inherent uncertainty surrounding the outcomes defines the behavior as risky [1]. For example, heavy drinking at a company party can lead to subjectively positive (e.g., feeling more at ease) or negative (e.g., behaving recklessly) consequences. Well-known risky behaviors (e.g., substance use, unprotected sex) can lead to negative impacts on morbidity and mortality [2, 3], but the risks of health behaviors involving medical procedures (e.g., taking medication, surgery) are less studied, although medical-related behaviors can have both positive and negative consequences. For instance, taking medication could lead to symptomatic relief or potential adverse effects [4].

Research indicates risk-taking propensity is influenced by the extent of the knowledge of all possible outcomes and by how much each outcome is valued by the risk-taker [1]. Importantly, individual characteristics also shape risk-taking [1]. For example, educational attainment may affect knowledge about potential outcomes due to variation in exposure to relevant information. Similarly, differences in the endorsement of numerous sociocultural values may influence risk-taking. For instance, collectivistic individuals [5] may shy away from socially discouraged behaviors (e.g., drinking heavily [6]) while also being more likely to engage in health-related risk-taking that benefits others (e.g., donating blood). The role of sociocultural factors in health-related risk-taking is understudied, particularly regarding medical risk-taking. As suggested by Weber and Johnson [7], uncovering the factors that contribute to health-related risk-taking will better inform health interventions.

Risk-taking is linked with multiple individual-level factors. For instance, longitudinal research indicates that age is related to health/safety risk-taking, with older individuals generally taking fewer risks [8]. Education is also linked to health-related risk-taking [9], perhaps due to better knowledge of the potential outcomes [7]. Research also suggests women report less risk-taking than men across many health behaviors [8]. For one, women are less likely to receive a flu vaccine [10], but on the other hand engage in more preventive medical care than men [11]. In addition, research suggests women are more likely than men to seek health-related information [12] and thus may be more inclined to educate themselves about the costs and benefits of medications, medical procedures, and so on. Studies typically examine gender in relation to health attitudes and behaviors using a dichotomous (i.e., male/female) variable; however some theories of gender suggest that it is beneficial to consider the degree to which individuals endorse gender-related traits that are considered masculine or feminine, which may be inconsistent with their identifications as male, female, or another gender [13]. Research examining the link between gender role orientation – rather than dichotomous gender identifications – and health-related risk-taking propensity across an array of health indicators is scarce, however.

Research suggests that, although genetic factors account for much of the variance across domains of risk-taking, decisions to engage in risk behavior  are also shaped by environmental factors [14]. Because of this, researchers recognize the need for more work examining potential sources that contribute to risk-taking propensity within specific domains [8]. To meet this need, the present study examines the role of several sociocultural factors with theoretical relevance to health behaviors.

## Sociocultural factors and health-related risk-taking

Norms transmitted through the sociocultural environment shape individual values [15]. These values then inform perceptions of – and engagement in – risky behavior [16]. However, literature examining the concurrent and incremental relations of relevant sociocultural values with risk-taking across health-related domains is scattered and scarce.

Some values that have received attention in the health literature are individualism and collectivism [5]. Broadly, individualistic-oriented people value independence and prioritize fulfilment of personal needs and goals. Conversely, collectivistic-oriented people value connection with others and may place others’ needs and goals above their own. Some research suggests collectivism is protective against several health/safety risk behaviors that may be socially discouraged by family and friends [6]. The same study found one positive relation between individualism and hazardous alcohol use [6].

Research on individualism and collectivism in medical risk-taking is limited. One study found a positive link between collectivism and perceived vulnerability to disease [17], indicating that people higher in collectivism may engage in medical behaviors that prevent disease or minimize its effects, perhaps so they do not burden close others. Additionally, some medical risks (e.g., blood donation) are predominantly altruistic in nature. In support of this, research from India – where collectivism is reputedly a dominant cultural value [18] – found that people are motivated to donate blood by a sense of community obligation [19].

Relatedly, some people value and are motivated by familial connections (i.e., familism) [20]. Although extant literature typically characterizes familism as a Hispanic cultural value, research suggests the construct can be applied across diverse ethnoracial groups [21]. There is little research examining the role of familism in health-related risk-taking among adults, aside from studies that examine familism in relation to alcohol and substance use, often finding an inverse link [22]. Because of the similarity between familism and collectivism [23], it is possible they may map onto health-related risk-taking similarly, with some exceptions. As with collectivism, familism may motivate individuals to engage in beneficial behaviors in order to preserve their own health and safety for the sake of their family. However, familism may relate to engagement in altruistic behaviors (e.g., blood donation) only when it directly benefits a family member. Because of the potential overlap between collectivism and familism on health-related risk-taking, it is important for research to disentangle their unique effects by including both factors within the same model.

Finally, health-related risk-taking may be shaped by one’s perceived control over their own health. Data suggest that 89% of the U.S. population believe in a God or God-like entity [24]. Many people believe that God is ultimately responsible for their health [25], a belief often referred to as the God Locus of Health Control (GLHC [26]). Research suggests increased GLHC may be linked to poor health behaviors (e.g., reduced vegetable consumption [27]). As with the constructs noted earlier, research examining GLHC and risk-taking across health domains is scarce. Given the theoretical relevance of each of these constructs, an investigation of their concurrent relations to health-related risk-taking is warranted.

## Current study

Studies examining how demographic and sociocultural values relate to both health/ safety related risk-taking (e.g., alcohol use, driving without a seatbelt) and medical-related risk-taking (e.g., donating blood, taking medication) within the same model are scarce. Risk-taking theorists posit that there are distinct psychological processes that are involved during risky decision making [1]. The thought processes underlying some risky decisions are characterized as either “hot” (i.e., affective) or “cold” (i.e., deliberate) [1]. However, the propensity, or inclination to engage in the health-related risk behavior may be further understood when contextualized within sociocultural perspectives and worldviews [28]. Researchers have noted the importance of accounting for demographic factors alongside sociocultural factors when examining health-related risk-behaviors (e.g., receiving a vaccine), so that groups of individuals who may be more or less likely to engage in specific health behaviors may be identified [28]. With this in mind, implementing a person-centered statistical approach (i.e., latent profile analysis; LPA), so that subtypes of such individuals may be identified, would also be beneficial. A previous study used a similar person-centered approach (i.e., Latent Class Analysis; LCA) to examine typologies of health behaviors (e.g., cigarette and alcohol use, vaccines and other routine medical care) among U.S. adults, and examined how four demographic factors (i.e., race/ethnicity, sex, region, and age) related to membership in each subtype [29]. Indeed, subtypes of individuals emerged during analysis that the researchers labeled “health promoting” (i.e., engaging in more healthy behaviors and fewer unhealthy behaviors) while other individuals were identified as “health compromising.” [29] This current study builds upon this past research in two very important ways.

1. Using LPA [30], we assessed health behaviors using a risk-taking framework. That is, we identified subtypes of individuals based on their propensity to engage in each health-related risk. Propensity, or inclination to engage in a health behavior, is a known predictor of engagement in the behavior. While LPA is a data-driven technique, based on previous research [29] we expected to identify a subtype of individuals characterized by higher propensity to engage in health benefiting behaviors (i.e., medical risks) and a simultaneous lower propensity to engage in health-harming behaviors (i.e., health/safety risks).
2. In addition to examining demographic factors related to membership in each subtype, we also assess the incremental role of the sociocultural factors described in the previous section (i.e., gender role orientation, individualism, collectivism, familism, GLHC). Due to noted gender differences in risk-taking [8], we further examined how gender moderated the relations between sociocultural factors and health-related risk-taking.

## Method

### Participants and procedure

The data were collected in November 2018 as part of a national study examining factors related to health and sickness behaviors among U.S. adults [31]. All procedures were approved by an Institutional Review Board (IRB), and all methods from this study were conducted in accordance with IRB guidelines. Questionnaires were administered by Qualtrics, a web-based survey platform. To be included, participants provided written informed consent, and: 1) were between 18 and 55 years old, 2) identified as non-Hispanic White, African American, or Hispanic, and 3) experienced sickness at some point during the past year. Of the 2815 people invited to participate, 1261 met inclusion criteria and elected to complete the survey.

### Measures

#### Health-related risk-taking

Two subscales from the Domain Specific Risk-Taking (DOSPERT-M^64^) measured health-related risk-taking. Six items were from a medical risk-taking subscale (e.g., “taking daily medication …” ; having “knee replacement surgery …” ) and five items were from a health/safety risk-taking subscale (e.g., “drinking heavily …” ; “driving a car without a seatbelt”). Items were measured on a scale of 1 (extremely unlikely) to 7 (extremely likely). Internal consistency values were good (medical α = .78; health/safety α = .83).

#### Demographic factors

Participants reported age using an open-ended response. We measured education level (7-point scale; 1 = some high school, 7 = doctorate); race/ethnicity (1 = non-Hispanic White, 2 = African American, 3 = Hispanic); annual income (12-point scale;1 = less than $10,000; 12 = more than $150,000); marital status (1 = currently married, 2 = previously married [i.e., widowed/separated/divorced], 3 never married); and gender (1 = male, 2 = female).

#### Sociocultural factors

##### Gender role orientation

Means were calculated for six items [32] assessing the degree to which participants viewed their attitudes/beliefs, appearance, and interests as either masculine or feminine on a 7-point scale (1 = very masculine, 7 = very feminine). Internal consistency was excellent (α = .97).

##### Individualism and collectivism

Means were calculated for six items measuring individualism (e.g., “Competition is the law of nature”) and eight items measuring collectivism (e.g., “The well-being of my co-workers is important to me”) using a 7-point scale [33] (1 = strongly disagree, 7 = strongly agree). Both subscales were internally consistent (individualism α = .82; collectivism α = .85).

##### Familism

Ten items assessed familism [34] (e.g., “When it comes to social responsibility, blood really is thicker than water”) with a 5-point scale (1 = strongly disagree, 5 = strongly agree) and were averaged for a total familism score. Internal consistency was excellent (α = .93).

##### God locus of health control (GLHC)

The summed score of six items assessed the extent participants felt God controlled their health [26] (e.g., “Most things that affect my health happen because of God”) using a 7-point scale (1 = strongly disagree, 7 = strongly agree). Internal reliability was excellent (α = .95).

#### Analytic strategy

Post-stratification weights were applied to survey data based on U.S. census reports of race/ethnicity, sex, and income. A latent profile analysis (LPA) was conducted with Mplus version 8.4 with full information maximum likelihood estimation using the health/safety and medical risk-taking items from the DOSPERT-M as indicators. LPA has become an increasingly popular analytic technique to assess the associations of concurrent health behaviors [35], and is advantageous over other cluster analytic techniques (e.g., hierarchical clustering) because it is model-based, allowing for researchers to more objectively assess how well the selected model fits the data [30]. In LPA, participants are grouped according to similarities across categories of a latent variable. Each participant is classified as a member of a single category. Categories are identified using data-driven techniques and guided by theoretical conceptualizations and the principle of parsimony. The process of identifying the categories (i.e., class enumeration) is informed by multiple indices [30], and adjusted according to recommendations specific to working with complex survey data [36]. As complex survey weights were applied to the data, we were guided by changes in the Bayesian Information Criterion (BIC), which indicates the probable odds of one model when compared to another model [37]. Lower BIC values are interpreted as better. Entropy, an indication of overall classification precision, was also inspected. Entropy scores can range from 0 to 1; higher scores reflect better classification precision [38]. In addition to assessing overall model entropy, we examined univariate entropy across each of the latent profile indicators. Univariate entropy is a measure of how well individual indicators can identify the profiles gleaned during class enumeration. Scores range from 0 to 1, with higher scores suggesting the indicator provides more information about the latent profiles than indicators with lower scores. This allowed us to further contextualize our profile selection [39]. Finally, interpretability, theoretical plausibility, and parsimony pertaining to the emergent latent categories also governed class enumeration decision-making.

Next, chi-square, ANOVA, and ANCOVA analyses assessed the demographic and sociocultural characteristics of profile members. ANOVA and ANCOVA analyses were interpreted using a Bonferroni adjusted *p*-value of < .006 to adjust for Type I error [40]. Dunnett C post-hoc pairwise comparisons were also conducted using a Bonferroni adjustment to account for unequal sample sizes across profile membership and violations of homogeneity of variance [41]. We then conducted follow-up a multinomial logistic regression to determine the incremental effects of demographic and sociocultural factors on group membership.

## Results

Sample weights were used when conducting all analyses. Two responses were omitted from the analysis for missing data necessary for computing sample weights (i.e., one case missing age data, the other missing sex data). See Table 1 for weighted descriptive statistics of the study’s sample. A zero-order correlation analysis indicated that all sociocultural factors were positively associated (*p* < .05), with the exception of gender role orientation and GLHC (n.s.).

**Table 1:** *Weighted sample characteristics*

Variable	Frequency (Percent)				
	Total Sample	Low Propensity	Moderate Propensity	High Propensity	Divergent Propensity
Gender					
Male	423(33.6)	**83(26.9)**	**98(37.9)**	**63(39.7)**	**178(33.6)**
Female	836(66.4)	**227(73.1)**	**161(62.1)**	**96(60.3)**	**351(66.4)**
Race/ethnicity					
non-Latinx White	856(68.0)	208(67.2)	173(66.6)	124(77.4)	351(66.4)
Latinx	229(18.20)	55(17.7)	49(18.9)	18(11.1)	108(20.3)
Black/African American	174(13.80)	47(15.1)	38(14.6)	18(11.5)	70(13.3)
Marital Status					
Married	508(40.3)	**105(33.8)**	**102(39.2)**	**87(54.5)**	**215(40.5)**
Formerly Married	205(16.3)	**53(17.2)**	**50(19.3)**	**17(10.5)**	**85(16.1)**
Never Married	546(43.3)	**152(49.0)**	**108(41.6)**	**56(35.1)**	**230(43.4)**
Variable	Mean (Standard Deviation)				
	Total Sample	Low Propensity	Moderate Propensity	High Propensity	Divergent Propensity
Age	37.02(12.49)	37.11(12.89)	36.58(12.41)	35.19(11.10)	37.73(12.67)
Income	5.62(3.73)	**5.77(3.83)**	**5.05(3.58)**^**a**^	**6.74(3.95)**^**ab**^	**5.46(3.62)**^**b**^
Education	3.56(1.51)	3.49(1.41)	3.52(1.55)	3.93(1.58)	3.52(1.53)
Gender role identity	4.71(1.82)	**5.01(1.69)**^**a**^	**4.51(2.01)**	**5.00(1.77)**	**4.55(1.79)**^**a**^
Individualism	5.82(1.72)	5.74(1.58)	5.59(1.89)	6.26(1.97)	5.86(1.60)
Collectivism	5.77(1.68)	5.69(1.69)	5.61(1.72)	6.23(1.90)	5.74(1.57)
Familism	3.83(.98)	3.77(1.07)	3.72(.95)	3.84(1.18)	3.91(.86)
GLHC	19.38(9.54)	18.02(9.46)	19.04(9.42)	23.06(10.83)	19.24(8.95)

*Note. N* = 1259. Weighted data presented; variables with significant omnibus frequency or mean differences across latent profiles highlighted in bold; means and standard deviation are unadjusted (adjusted means reported in text); superscripts indicate significant pairwise comparisons along each row

### Latent profile analysis (LPA)

**Fig. 1:**
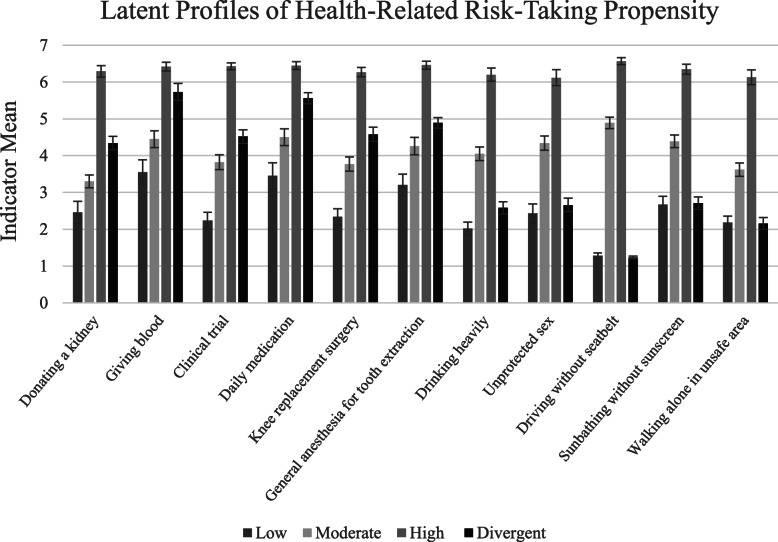
Weighted means from Mplus LPA reported here. Low propensity *n* = 305, moderate propensity *n* = 235, high propensity *n* = 154, divergent propensity *n* = 565

Using BIC, entropy, and interpretability of profiles, a four-profile model was selected. See Table 2 for numeric class enumeration details. Specifying a five-profile solution yielded a warning that the best loglikelihood value could not be replicated, indicating five profiles were too many to extract from these data. Clear patterns of responses emerged across the four profiles (Fig. 1). While all subtypes were characterized by generally higher endorsements of medical risk-taking than health/safety related risk-taking, with few exceptions participants grouped themselves as low (*n* = 305, 24.24%), moderate (*n* = 235, 18.63%), or high (*n* = 154, 12.26%) propensity risk-takers. One profile emerged (*n* = 565, 44.88%) that stood out from the other three, with a noticeably steeper difference across mean endorsements of medical indicators and health/safety indicators. Profiles were labeled 1) low propensity, 2) moderate propensity, 3) high propensity, 4) divergent. The highest univariate entropy score was .611 (item: propensity to drive without a seatbelt) and the lowest univariate entropy score was .182 (item: propensity to opt for general, rather than local anesthesia for a wisdom tooth extraction). The remaining indicators’ univariate entropy scores fell between .201 and .260.

**Table 2:** *LPA enumeration indices*

	AIC	BIC	aBIC	Entropy
1	59,914.74	60,027.78	59,957.90	–
2	57,164.73	57,339.42	57,231.42	.94
3	56,373.43	56,609.78	56,463.67	.82
4	55,134.84	55,432.85	55,249.61	.89

*Note.* AIC = Akaike’s Information Criterion; BIC = Bayesian Information Criterion; aBIC = adjusted BIC

### Demographic analysis

Chi-square analyses indicated that gender was related to profile membership (χ^2^[*N* = 1257, 3] = 11.15, *p* = .011; ϕ = .09). A higher proportion of women (27.20%) than men (19.2%) were low propensity profile members, while a higher proportion of men than women were members in both the moderate (men = 23.2%; women = 19.3%) and high (men = 14.9%; women = 11.5%) propensity profiles. Divergent profile membership was equivalent across men and women (approximately 42% each). Marital status was also related to profile membership (χ^2^[*N* = 1260, 6] = 20.86, *p* = .002; ϕ = .13). Nearly identical proportions of individuals from each marital status group identified as divergent profile members (approximately 42%), with proportional discrepancies occurring in both the low and high propensity profiles. For the low propensity profile there were fewer currently married (20.6%) than formerly married (25.9%) and never married (27.8%) participants. For the high propensity profile, there were more married (17.1%) than never married (10.3%) and formerly married (8.3%) participants. Race/ethnicity was unrelated to profile membership (*p* = .142).

ANOVA analyses indicated the average age of participants did not differ across profiles, (*p* = .234). Mean income was higher for members of the high (*M* = 7.01, *SD* = 3.80) and low (*M* = 6.03, *SD* = 3.88) groups than for members of the moderate (*M* = 5.13, *SD* = 3.65) group. Income was also higher in the high propensity profile than the divergent propensity profile (*M* = 5.59, *SD* = 3.94), *F*(3,1254) = 8.84, *p* < .001; η_p_^2^ = .02. Further, education level was significantly different across profiles, *F*(3,1172) = 4.16, *p* = .006. Given the strong conceptual relation between income and education, we conducted a follow-up ANCOVA controlling for income, which rendered the effect of education level non-significant, (*p* = .116).

### Sociocultural analysis

Factorial ANCOVAs assessed the mean differences of each sociocultural factor in terms of profile membership. Each model included gender, marital status, and income, as well as each sociocultural factor not specified as the dependent variable as covariates to better assess the incremental and unique relations between profile memberships and the sociocultural factor of interest. We describe the main effects of both profile and gender, and the interaction effect between the two variables, below.

There was a difference in gender role orientation in terms of both profile [*F*(3,1161) = 5.17, *p* = .002; η_p_^2^ = .01] and gender [*F*(1,1161) = 466.77, *p* < .001; η_p_^2^ = .29]. Low propensity profile members endorsed gender role orientation significantly higher (i.e., orienting toward femininity) than divergent profile members; no other pairwise differences were found. Women scored significantly higher than men on gender role orientation. The interaction between profile and gender was nonsignificant, (*p* = .068).

There were no differences in individualism across propensity profiles [*F*(3,1161) = 1.90, *p* = .129]. Men endorsed individualism more than women, *F*(1,1161) = 39.32, *p* < .001; η_p_^2^ = .03, although there was no interaction between profile and gender (*p* = .304). Regarding collectivism, no differences were detected across propensity profiles (*p* = .788), gender (*p* = .213), or the interaction between the two variables (*p* = .911).

For familism, the *F* statistic for profile (*p* = .014) and gender (*p* = .040) did not pass the conservative corrective threshold of .006. The interaction between gender and profile was nonsignificant (*p* = .437). GLHC differences across profiles approached significance (*p* = .008), and men endorsed GLHC more than women, *F*(1,1161) = 12.77, *p* < .001; η_p_^2^ = .01). However, the interaction between profile and gender was nonsignificant GLHC (*p* = .066).

### Follow-up multinomial regression

Because the divergent profile showed a notable uncoupling of health/safety risk-taking items and medical risk-taking items, whereby the former was endorsed much less than the latter, we conducted a follow-up multinomial logistic regression to examine characteristics of divergent profile members compared to other profiles’ members. For a detailed exploration, we included all demographic and sociocultural factors described earlier and set significance at *p* < .050.

Numeric results are in Table 3. Compared to low propensity profile membership, there was a greater probability for divergent profile members to 1) have lower income, 2) be married (relative to being never married), 3) orient toward masculinity, and 4) endorse more familism. Compared to moderate propensity profile membership, there was a greater probability for divergent profile members to endorse more individualism and familism. Compared to high propensity profile membership, there was a greater probability for divergent profile members to 1) be older, 2) be White, as compared to Hispanic, 3) orient toward masculinity, 4) endorse less GLHC, and 4) endorse more familism.

**Table 3:** *Coefficients from multinomial logistic regression model*

	Low vs. Divergent Propensity				Moderate vs. Divergent Propensity				High vs. Divergent Propensity			
Variable	***B***	***SE***	***p***	***OR***	***B***	***SE***	***P***	***OR***	***B***	***SE***	***p***	***OR***
Age	−.01	.01	.224		−.01	.01	.057		**−.04**	**.01**	**<.001**	**.97**
Education	−.05	.06	.46		.06	.07	.373		.00	.08	.961	
Income	**.07**	**.03**	**.011**	**1.07**	−.04	.03	.150		.04	.04	.273	
Married	**−.27**	**.19**	**.048**	**.69**	.17	.20	.397		.30	.25	.231	
Formerly Married	.01	.23	.970		.42	.24	.085		.09	.34	.803	
Gender (Male)	.16	.22	.468		.57	.23	.013		.34	.26	.190	
Latinx	−.15	.21	.459		−.11	.21	.619		**−.79**	**.30**	**.008**	**.453**
African American	.20	.22	.377		.02	.24	.948		−.24	.30	.783	
Gender Role Orientation	**.19**	**.06**	**.001**	**1.21**	.09	.06	.134		**.18**	**.07**	**.009**	**1.19**
Individualism	−.05	.06	.385		**−.12**	**.06**	**.033**	**.883**	.02	.08	.793	
Collectivism	.02	0.06	.720		.07	.07	.320		.07	.09	.459	
Familism	**−.20**	**.08**	**.019**	**.821**	**−.21**	**.09**	**.018**	**.810**	**−.35**	**.11**	**.001**	**.71**
God Locus of Health Control	−.01	.01	.174		.00	.01	.948		**.03**	**.01**	**.009**	**1.03**

*Note. N* = 1259. Weighted data presented. Divergent profile reference for all other profiles. Significant effects highlighted in bold. Exp(B) (odds ratio) presented for significant effects only. The reference group for married and formerly married is never married. The reference group for male is female

The reference group for both Hispanic and African American is non-Hispanic White.

## Discussion

### LPA findings

For three profiles, responses appeared to map onto a general propensity for risk-taking (low, moderate, and high), particularly among the health/safety items. There were some notable similarities across profiles. For instance, across low, moderate, and divergent profiles, more invasive medical risk behaviors were generally endorsed lower than less obviously invasive medical risk behaviors. Moreover, the least endorsed invasive medical items were comprised of behaviors that potentially benefited others more than oneself (e.g., donating a kidney). There were similar trends across profiles for health/safety-related risks, particularly across the low and divergent propensity profiles, and both endorsed driving without a seatbelt the least. For these participants, perhaps the potential adverse consequences of driving without a seatbelt (e.g., serious injury) serve as a deterrent. There were additional similarities across the low and moderate profiles for kidney donation, blood donation, and participating in a clinical trial, which could arguably be perceived as entirely altruistic when compared to other Univariate scores suggested that the propensity to not wear a seatbelt was the strongest identifier of all of the profiles while the propensity to opt for general, rather than local anesthesia for a wisdom tooth extraction was the weakest individual identifier of the profiles. It is possible that this latter item’s low univariate entropy score was due to a nuance in the wording of the item as compared to the other indicators. Specifically, this item asks participants to report their propensity to engage in a health-related risk behavior (i.e., choice of anesthesia) *after* another health-related risky decision has already been made (i.e., tooth extraction). Asparouhov and Muthén [39] indicate that items with scores that are nearing zero may be omitted from the model as they do not contribute substantial information about the characteristics of the profiles. We elected to retain this item in the interest of preserving all health-related indicators of the DOSPERT-M to better inform future researchers examining health-related risk-taking with this measure. Univariate entropy among the remaining indicators were modest. In the context of the relatively high model entropy, it is suggestive that the collection of items is more important for identifying profiles than singular indicators.

### Profile characteristics

Results suggested being married was associated with generally high risk-taking while being never married was associated with generally low risk-taking. Research suggests marriage may serve as either a risk or protective factor across a range of health behaviors [42]. Married people may be more likely to have health insurance [42], which could contribute to a greater willingness to engage in health-related risk-taking. Other research suggests that married couples influence their spouses to limit behaviors that may compromise health [43], and that spousal health risk behaviors positively correlate with one another [44]. This may explain the current findings, though spousal risk-taking was not measured in this study. Also, high propensity profile members were more likely to have a higher income than moderate and divergent profile members. This is consistent with literature suggesting that individuals with higher incomes have more access to – and may be more likely to seek – medical care [45], and other literature connecting higher income levels with more engagement in some harmful health behaviors (e.g., hazardous alcohol use and related behaviors) [46]. With this in mind, it is possible that the potential adverse health consequences associated with some of health-related risks may be perceived as less of a hindrance for those who can afford medical care.

Aligned with theory that suggests health-risk behaviors may be part of an overarching gendered script [47], men were more likely than women to be members of higher health/safety risk profiles. Similarly, orienting toward femininity related to a higher probability of being a generally low risk-taker. Previous research suggests that men view potential health-related hazards as substantially less risky and are more inclined to participate in health-related risk activities than women [48]. Risk-taking has been described as part of a hegemonic masculine gender role that is prevalent in Western societies and a potential source of status among other men [49]. Connell & Messerschmidt [49] note that gender is a relative construct; thus, it is also possible that refraining from risk-taking may be viewed as part of a feminine gender role, as suggested in previous research [50].

### Factors predicting divergent profile membership

The divergent profile exhibited the clearest differentiation between lower endorsements of health/safety-related risk-taking and higher endorsements of medical-related risk-taking. Data showed married participants were more likely to be divergent, rather than low, propensity profile members compared to never married participants. As expected from research that suggests risk-taking declines with age [8], older participants were more likely than younger participants to be members of the divergent profile than the high-risk profile. It is possible that older adults in our sample perceived medical care more favourably than younger adults [51]. In addition, Hispanics were more likely than non-Hispanic Whites to be members of the divergent rather than the high propensity profile, aligning with research indicating that Hispanics view potentially hazardous health behaviors as more risky than non-Hispanic Whites [48].

One seemingly counterintuitive finding suggested divergent profile members had lower incomes than low propensity profile members, indicating that lower income predicted higher medical risk-taking. It is possible this connection may be a function of uncertain future environments. That is, individuals in unpredictable or harsh environments (e.g., lower socioeconomic circumstances [52]) may gravitate toward risky behaviors, particularly those with immediate benefits and future costs [53]. Medical risk-taking may fit this pattern. For example, present relief from allergy symptoms may outweigh future costs of long-term antihistamine use.

Consistent with the ANCOVA findings, femininity was related to low, rather than divergent, profile membership; however, results also showed that femininity was related to high, rather than divergent, profile membership, and contrasts gender role theories previously described. It is feasible that this is a limitation of measurement. Participants in this study rated themselves along a continuum as masculine or feminine. The extant gender role research highlights the complex and multi-dimensional nature of gender roles, and the importance of accounting for these nuances when predicting health behaviors [54].

Surprisingly, individualism but *not* collectivism predicted divergent profile membership. Individualistic-oriented individuals were more likely to engage in medical risk-taking but less likely to engage in health/safety risk-taking than people who generally endorse moderate levels of risk-taking. Theory suggests individualistic people prioritize personal goals and responsibilities [55]. Our findings indicate that the inclinations to refrain from potentially harmful health behaviors while also engaging in potentially beneficial health behaviors may be an enactment of individualistic values, perhaps as a means of self-preservation. The null effects of collectivism were unexpected, given research linking collectivism to health behaviors [6]. As measured in this study [33], collectivism captured participants’ values related to several groups (e.g., family, co-workers), which may have contributed to the lack of collectivism-related findings in these results. If the group that a person feels connected to informs their perceptions of risk-taking, more specificity about the group may be needed.

Importantly, familism predicted membership in the divergent profile compared to all other profiles. Like collectivism, familism emphasizes interdependence [34], though exclusively in relation to family. Previous research links familism to better physical and subjective health [56, 57], and to less engagement in health/safety risk behaviors [22]. As many of the health/safety related risks may be perceived as more hazardous than many of the medical risks, familism-oriented individuals might be more likely to engage in medical, but not health/safety, risk-taking. Researchers have also linked familism to prosocial tendencies to help others [58], which may contribute to the willingness to engage in medical risks that do not directly preserve the self (e.g., kidney donation).

Finally, lower GLHC was related to membership in the divergent profile as compared to the high-risk profile. Some research suggests that assigning control of one’s health to an outside source reduces one’s ability to accurately perceive risk [59]. Additionally, research indicates that people who assign locus of control to an external source may not be inclined to seek out risk-related information [60]. In this case, not having as much information about the potential adverse effects of engaging in health-related risk-taking may render people with higher GHLC more comfortable with such risks.

### Strengths, limitations, and future directions

This study must be interpreted considering some limitations. Data were cross-sectional and cannot address directionality. In addition, the non-convergence of the LPA solution with five profiles may reflect insufficient data points to support five subtypes and does not necessarily indicate that five or more profiles do not exist. Examining this study’s research questions with larger samples would be beneficial to assess the veracity of this potential limitation.

Criteria for the larger project from which these data are derived required that participants reported experiencing an illness in the past year, although there were no restrictions on illness severity. Although adults in the U.S. experience an average of two to three colds annually [61], these findings may not generalize to those individuals who had not been sick during the previous year. Relatedly, we did not account for individual health of participants or the health-related behaviors they currently engaged in (e.g., current medications), although these factors may influence how likely they are to endorse some of the health variables measured in this study. Also, the effect sizes reported in this study were generally modest and suggest that future research should model sociocultural factors as upstream of more proximal mechanisms to risk-taking within a more comprehensive framework. For instance, researchers could use a mixed methods approach to identify the reasons that individuals find these health behaviors risky (e.g., what consequences do they anticipate), and then link these factors to those risk perceptions. Furthermore, in the interest of having a large enough sample to analyze differences in health outcomes across race and ethnicity, we only included U.S. adults who identified as either White, Black, or Hispanic, which limits the ability of this study to generalize to U.S. adults from other racial and ethnic backgrounds. Also, though analyzing gender as both a label (i.e., male/female) and as an orientation (i.e., masculine/feminine) was a strength of the study, a binary perspective on gender does not adequately address the complex gender identities experienced by people in many parts of the world.

Although it is beyond the scope of the current study to examine how the link between sociocultural values and health-related risk-taking profiles varied as a function of multiple key demographic factors, there is a strong practical and theoretical rationale for doing so. For example, older adults’ perspectives are shaped by experiences and cognitive functioning that is markedly different than those of younger adults [62]. While the current study found links between age and risk-taking propensity that mapped onto past research, future research that examines how age moderates the relationship between the independent and dependent variables of interest in this study be of even further benefit for increasingly targeted public health programming.

## Conclusion

Electing to engage in any health behavior, whether the behavior is medically recommended or not, is characterized by risk. The decision to engage in specific health behaviors then depends upon, among other things, individuals’ assessments of the potential consequences of the behavior. These assessments include both the probabilities and the valence of the respective consequences. For health-related risk behaviors such as donating blood, a person may weigh the possibility of physical discomfort of the needle against the possibility of experiencing psychological satisfaction from assisting someone in need. For health-related risk behaviors such as heavy alcohol use, a person may weigh the possibility of long-term cirrhosis of the liver against the possibility of short-term relief from stress. Perceptions of the consequences associated with each health behavior thus play a major role in shaping the willingness of a person to engage in health-related risk-taking.

By examining health-related risk-taking propensity across both medical risks and health/safety risks using a person-centered approach, this study aligns with previous research suggesting that there are subtypes of individuals who can be considered “health-promoting.” [29] Whereas previous research has found these health-promoting subtypes in regards to frequency of specific types of health behavior [29], this current study suggests that similar groups of people can also be identified by their propensity to engage in such behaviors. It is important to consider that the context that underlies the decision to engage in the risk behavior at the moment of the behavior is unique [1] (e.g., the careful and long-term planning of an extensive medical procedure as compared to the decision to have sexual intercourse without a condom). We cannot know the extent to which the propensity that individuals in this study are endorsing map onto their current or later behavior. However, an individual’s assessment of their own inclination to engage in each of these behaviors may be shaped by common upstream factors, and there is supporting research that indicates this inclination may predict later actual engagement in the behavior [63]. As a novel contribution to the literature, this current study shed light on the sociocultural values that individuals who are likely to engage in health benefiting behaviors ascribe to, above and beyond more often studied demographic characteristics. In the interest of both public and personal health, it is imperative that health experts understand potentially modifiable characteristics that may shape peoples' inclinations to engage in health behaviors that benefit themselves and others, as well as inclinations to refrain from health behaviors that are generally hazardous. This study demonstrated that a subtype of people meeting this specific pattern of health protection can be identified. As such, this study is an important step toward refining health programs and materials designed to protect and improve personal and public health.

## Data Availability

The datasets used during the current study are available from the corresponding author on reasonable request.
